# Nutritional assessment and prognosis of oral cancer patients: a large-scale prospective study

**DOI:** 10.1186/s12885-020-6604-2

**Published:** 2020-02-22

**Authors:** Xiaodan Bao, Fengqiong Liu, Jing Lin, Qing Chen, Lin Chen, Fa Chen, Jing Wang, Yu Qiu, Bin Shi, Lizhen Pan, Lisong Lin, Baochang He

**Affiliations:** 1 Department of Epidemiology and Health Statistics, Fujian Provincial Key Laboratory of Environment Factors and Cancer, School of Public Health, Fujian Medical University, Fujian, China; 2 Key Laboratory of Ministry of Education for Gastrointestinal Cancer, Fujian Key Laboratory of Tumor Microbiology, Fujian Medical University, Fujian, China; 3 Laboratory Center, The Major Subject of Environment and Health of Fujian Key Universities, School of Public Health, Fujian Medical University, Fujian, China; 4 Department of Oral and Maxillofacial Surgery, the First Affiliated Hospital of Fujian Medical University, Fujian, China

**Keywords:** Serum albumin, Body mass index, Prognostic nutritional index, Oral cancer, Prognosis

## Abstract

**Background:**

To evaluate and compare the prognostic performance of four nutritional indicators body mass index (BMI), serum albumin (ALB), prognostic nutritional index (PNI) and nutritional risk index (NRI) in oral cancer patients, and to predict the response to chemotherapy in patients with different nutritional status.

**Methods:**

This prospective study which involved 1395 oral cancer patients was conducted in Fujian, China from September 2007 to November 2018. The BMI, PNI and NRI were calculated according to the following formulas: BMI = weight / height^2^ (kg/m^2^), PNI = albumin (g/l) + 0.005 × lymphocyte (count/μl) and NRI = (1.519 × albumin, g/l) + (41.7× present/ideal body weight), respectively. The univariate and multivariate Cox proportional hazards models were used to compare the prognostic value of BMI, ALB, PNI and NRI in overall survival (OS) in oral cancer.

**Results:**

Patients with BMI < 18.5 kg/m^2^ (VS 18.5 kg/m^2^ ≤ BMI < 24 kg/m^2^) had a poor survival outcome (HR = 1.585; 95% CI: 1.207–2.082 ). ALB, PNI, NRI were inversely correlated with OS of oral cancer (HR = 0.716; 95% CI: 0.575–0.891; HR = 0.793; 95% CI: 0.633–0.992; HR = 0.588; 95% CI: 0.469–0.738, respectively). In addition, the prognostic predictive performance of NRI was superior to BMI or ALB or PNI. Interestingly, compared with patients with better nutritional status, chemotherapy was significantly associated with poorer OS in malnourished oral cancer patients.

**Conclusions:**

BMI, ALB, PNI and NRI are of prognostic value in patients with oral cancer and the prognostic performance of NRI was superior to BMI or ALB or PNI. Malnutrition (BMI < 18.5 kg/m^2^ or ALB< 40 g/l or PNI < 49.3 or NRI < 97.5) could predict an unfavorable response to chemotherapy in oral cancer patients.

## Background

Oral cancer is one of the most common malignancies in head and neck, with an increasing incidence globally and has been a major public health problem in developing countries [1, 2]. The prognosis of oral cancer patients has not been obviously improved and remains relatively poor with an overall 5-year survival rate of approximately 50% [3, 4].

Malnutrition, a subacute or chronic state in cancer patients, may impair immune function, increase susceptibility to infection and complications of the treatment, thus leading to an increased mortality of cancer patients [5, 6]. In general, oral cancer patients go through odynophagia and dysphagia, and may undergo chronic fatigue which increased risk of malnutrition [7]. Published reports indicated that almost 30% of oral cancer patients suffered from malnutrition that be associated with adverse survival rates [7, 8]. Hence, assessment of nutritional status is critical to the prognosis of oral cancer patients. Nevertheless, clinical assessment of malnutrition of oral cancer patients is often neglected.

Traditional nutritional indicators such as body mass index (BMI) and serum albumin (ALB) have been widely used in clinical to assess the nutritional status of the cancer patients and have been shown to be associated with the prognosis of a variety of tumors such as rectal cancer, head neck, oral cancer and gynecologic malignancies [9–13]. In addition to BMI and ALB, two comprehensive indicators prognostic nutritional index (PNI) which includes albumin and lymphocyte and nutritional risk index (NRI) which combines weight, height and serum albumin levels, have also been reported as simple but sensitive methods that can objectively assess the nutritional status of cancer patients and predict their prognosis in esophageal squamous cell carcinoma, liver cancer, lung cancer, and gastric cancer et al. [11, 14–19]. However, there are few studies comprehensively explored the relationship between different nutritional indicators and prognosis of oral cancer patients.

Therefore, we choose the four most commonly reported indicators BMI, ALB, PNI, NRI and conducted a large scaled prospective study of oral cancer patients to primarily assess the association between malnutrition and mortality using four indicators (ALB, BMI, PNI and NRI), and to compare the prognostic performance of ALB, BMI, PNI and NRI. Additionally, we evaluated the association between survival and chemotherapy in patients with different nutritional status.

## Methods

### Study subjects

From September 2007 to November 2018, oral cancer patients were consecutively recruited from The First Affiliated Hospital of Fujian Medical University (Fujian, China). The inclusion criteria were as follows: 1) All cases were newly diagnosed with primary oral cancer that was confirmed by histology; 2) all cases were 20–80 years old. Those who had recurrent oral cancer, metastatic cancer, previous chemotherapy or radiotherapy were excluded. Besides, patients with incomplete information on height or weight or albumin levels were also excluded. Finally, the analysis dataset included 1395 patients with oral cancer.

Demographic data (age, sex, education levels, occupation, origin, height and weight) and clinical characteristics (clinical classification, histological types, tumor location, therapeutic regimen) were collected from medical records. All patients were followed up through telephone interview or by checking medical records of readmission. Telephone interview was conducted at 6-month intervals until death or the last follow-up. The endpoint was overall survival (OS) which was calculated from the date of diagnosis to the date of death from any cause. The informed consents were obtained from all patients. This study was approved by the institutional review board of Fujian Medical University (Fuzhou, China) and performed according to the ethical standards described in the Declaration of Helsinki.

### Variables definitions

All the four indicators were collected at the time of diagnosis. The BMI formula used was as follows: weight / height^2^ (kg/m^2^). Malnutrition was defined by a BMI < 18.5 kg/m^2^, while overweight or obese was defined if the BMI ≥24 kg/m^2^. ALB (g/l) is a commonly used biochemical markers of which clinical recommend range is 40–55 g/l. Malnutrition was defined by ALB < 40 g/l, while normal nutrition status was defined by 40 ≤ ALB < 55 g/l. The PNI was calculated using the formula: albumin (g/l) + 0.005 × lymphocyte (count/μl), the original formulas and definition of PNI is come from Onodera et al. [20]. Patients were divided into well-nourished (PNI < 49.3) and malnourished group (PNI ≥49.3) according to the median value of PNI. The NRI was calculated according to the formula: NRI = (1.519 × albumin, g/l) + (41.7× present/ideal body weight), the original formulas and definition of NRI is come from Buzby et al. [21]. The ideal body weight was computed based on the Lorenz equation: For males: Height - 100 - [(Height - 150)/4], and for females: Height - 100 - [(Height - 150)/2.5]. Malnutrition was defined by NRI < 97.5, while normal nutrition status was defined by NRI ≥97.5 [11, 22]. For data analysis in this study, we updated the staging of cases before 2010 based on pathology reports according the 7th edition of AJCC, so all the patients were staged according to the 7th edition of AJCC pathological staging system (2010) to make all the date comparable.

### Statistical analyses

Survival analysis was performed by using the Kaplan-Meier log-rank test. Hazard ratios (HRs) and 95% confidence intervals (CIs) were calculated by univariate and multivariate Cox regression models. Akaike information criterion (AIC), Harrell concordance index (C-index), Somer’s D and Likelihood Ratio χ^2^ (LR χ^2^) were used to evaluate the discriminational power of the multivariate regression models including different nutritional markers. The higher C-index or Somer’s D or LR χ^2^, the better predictive power of regression models. The lower AIC value, the better model fit. All statistical analyses were performed using the statistical software package R (version 3.1.1). Statistical significance was reported at *P* < 0.05.

## Results

A total of 1395 oral cancer patients were enrolled in the study. Among them, the mean age was 57.23 years (range 20–80 years, SD 13.80 years), and the ratio of males to females was 1.7:1 (878/517). Patients with T_4_ and N_0_ disease formed the most common T and N classification, respectively. The majority of the patients (78%) had a history of surgical therapy, while 635 (45.52%) did not receive adjuvant therapy. The overall 5-year survival rate was 68.48% (95%*CI*: 0.65–0.71). Details of demographics and clinical characteristics of all patients were listed in Table 1. For all patients, mean BMI was 22.2 kg/m^2^ (range 10.7–45.4 kg/m^2^). 343 patients (24.59%) presented a BMI ≥24 kg/m^2^ (overweight or obese), whereas 214 subjects (15.34%) with BMI < 18.5 kg/m^2^ (underweight). At diagnosis, the median value of ALB, PNI and NRI were 40.5 (range: 3.9 to 72.3), 49.3 (range: 11.6 to 81.5) and 102.9 (range: 49.9 to 156.1), respectively as results shown in Table 2.

**Table 1:** Demographics and univariate survival analysis results of all patients

Characteristic	No (%)	Univariate analyses	
		log-rank *P*	*HR*(95%*CI*)
Gender		0.022	
male	878 (62.94)		1.000
female	517 (37.06)		0.774 (0.621,0.964)
Age (years)			
< 55	556 (39.86)		1.000
≥ 55	839 (60.14)		1.677 (1.338,2.101)
Occupation		0.021	
farmer	423 (30.32)		1.000
worker	232 (16.63)		0.788 (0.589,1.056)
office worker and other	740 (53.05)		0.724 (0.574,0.913)
Origin		0.055	
urban area	585 (42.12)		1.000
rural area	804 (57.88)		1.230 (0.995,1.521)
Education level		< 0.001	
Illiteracy	92 (6.93)		1.000
Primary-middle school	967 (72.87)		1.316 (0.826,2.096)
High school and above	268 (20.20)		0.663 (0.383,1.147)
TNM classification		< 0.001	
I	178 (14.90)		1.000
II	308 (25.77)		1.395 (0.870,2.234)
III	219 (18.33)		2.134 (1.319,3.453)
IV	490 (41.00)		2.710 (1.767,4.158)
pT classification		< 0.001	
T_1_	217 (18.19)		1.000
T_2_	395 (33.11)		1.316 (0.897,1.930)
T_3_	178 (14.92)		1.734 (1.124,2.676)
T_4_	403 (33.78)		2.205 (1.531,3.175)
pN classification		< 0.001	
N_0_	839 (69.17)		1.000
N_1_	186 (15.33)		2.200 (1.638,2.954)
N_2_	181 (14.92)		3.392 (2.600,4.423)
N_3_	7 (0.58)		5.016 (2.214,11.367)
Pathological grading		0.011	
well	558 (51.67)		1.000
moderate	375 (34.72)		1.459 (1.137,1.872)
poor	147 (13.61)		1.253 (0.867,1.811)
Adjuvant therapy		0.064	
NO	635 (48.03)		1.000
RT	193 (14.60)		0.851 (0.601,1.205)
CT	197 (14.90)		1.367 (1.019,1.834)
CRT	297 (22.47)		1.144 (0.880,1.488)
Tumor site		0.044	
Tongue	525 (37.91)		1.000
Gingiva	155 (11.19)		1.351 (0.981,1.860)
Floor of mouth	90 (6.50)		1.404 (0.933,2.114)
Palate	103 (7.44)		1.036 (0.675,1.590)
Buccal	180 (13.00)		1.148 (0.832,1.585)
Others	332 (23.97)		0.798 (0.591,1.077)
Surgery therapy		< 0.001	
No	156 (11.45)		1.000
Yes	1207 (88.55)		0.194 (0.152,0.249)

**Table 2:** Association between BMI, ALB, PNI and NRI and overall survival of oral cancer patients

variables	Total number (%)	Number of Censored (%)	Number of death (%)	log-rank *P*	*HR* (95%*CI*)
BMI (kg/m^2^)				0.001	
18.5–23.9	838 (60.07)	628 (60.44)	210 (58.99)		1.000
< 18.5	214 (15.34)	138 (13.28)	76 (21.35)		1.462 (1.124,1.902)
≥ 24.0	343 (24.59)	273 (26.28)	70 (19.66)		0.819 (0.625,1.074)
ALB (g/l)				< 0.001	
< 40.0	613 (43.94)	419 (40.33)	194 (54.49)		1.000
≥ 40.0	782 (56.06)	620 (59.67)	162 (45.51)		0.547 (0.444,0.674)
PNI				< 0.001	
< 49.3	703 (50.39)	488 (46.97)	215 (60.39)		1.000
≥ 49.3	692 (49.61)	551 (53.03)	141 (39.61)		0.632 (0.511,0.783)
NRI				< 0.001	
< 97.5	388 (27.81)	247 (23.77)	141 (39.61)		1.000
≥ 97.5	1007 (72.19)	792 (76.23)	215 (60.39)		0.495 (0.400,0.612)

**Fig. 1:**
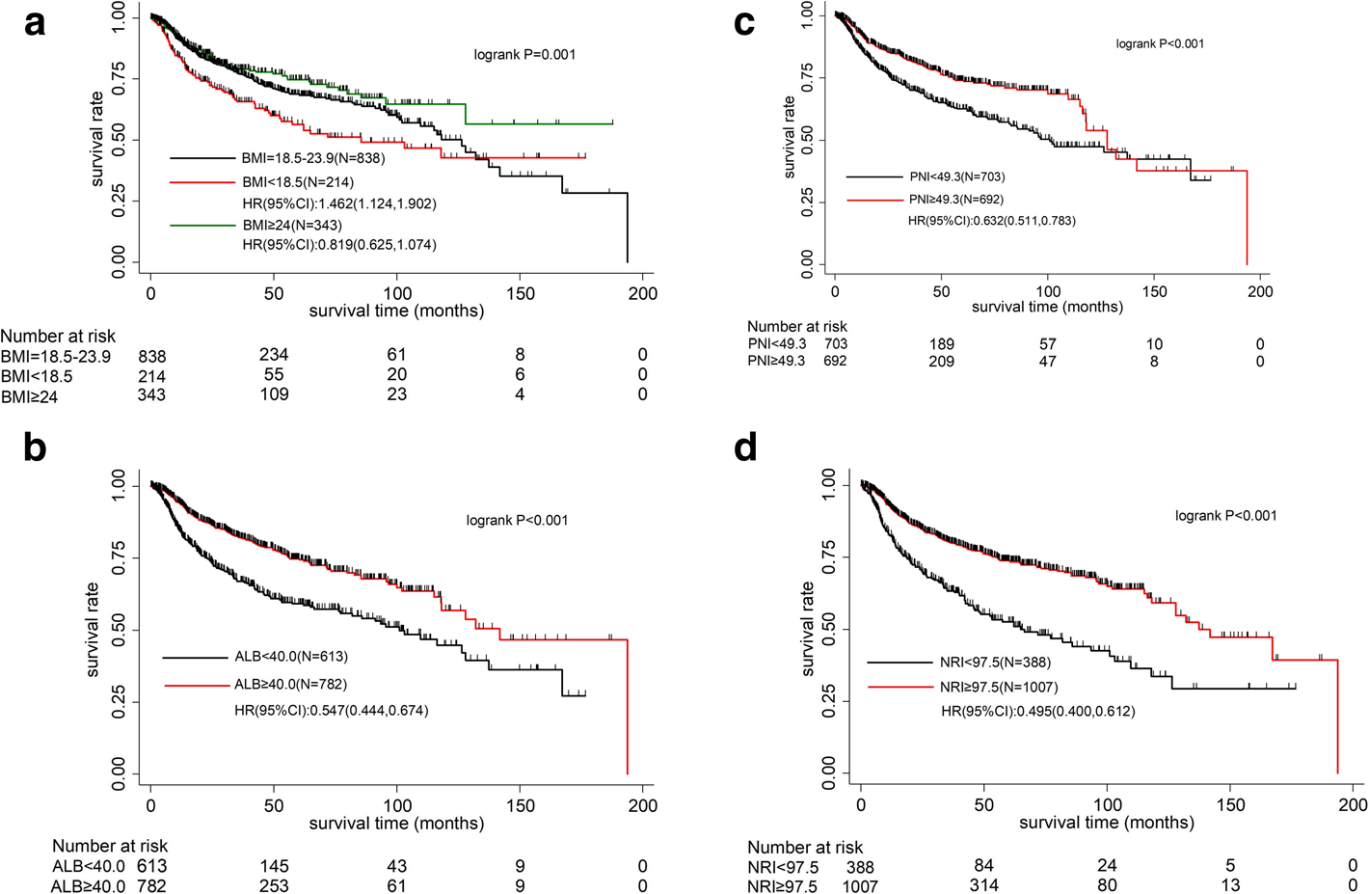
**a** Overall survival according to body mass index (BMI). **b** Overall survival according to serum albumin (ALB). **c** Overall survival according to prognostic nutritional index (PNI). **d** Overall survival according to nutritional risk index (NRI)

Univariate analysis and log rank test were performed and associations between BMI, ALB, NRI, PNI and OS of oral cancer patients were listed in Table 2. Worse OS was observed in patients with a BMI lower than 18.5 kg/m^2^. However, there was no significant different in OS between patients with BMI > 24 kg/m^2^ and those with normal weight (Fig. 1a). Moreover, ALB, PNI and NRI were inversely correlated with the prognosis of oral cancer patients (all *P* < 0.001, Fig. 1b-d).

Next, multivariate Cox regression analysis was applied. Considering the potential collinearity problem, we performed correlation analyses among four nutritional indexes and results were listed in Additional file 1: Table S1. Varied extents of correlation were observed among these four nutritional indexes, relative strong correlation exists between PNI and ALB (r = 0.7988), NRI and BMI (r = 0.7638), NRI and ALB (r = 0.7246), NRI and PNI (r = 0.649). Weak correlation exists between ALB and BMI (r = 0.1719), PNI and BMI (r = 0.2263). Since there are correlations among the four nutritional indexes, we put these indexes in multivariate Cox regression analysis respectively and build different regression model as shown in Table 3. With regard to the variable to be adjusted in the multivariate cox regression model. We analyzed the correlation between nutritional indicators and clinically relevant variables, results of which are listed in Additional file 1: Table S2. Nutritional indicators were widely associated with cancer-associated variables such as clinical classification and treatment. Therefore, we adjusted cancer-associated variables to exclude the confounding effects of these variables on oral cancer prognosis in multivariate Cox regression analysis. After adjusting for age, gender, occupation, education level, residence, clinical classification, pathological grading, surgery therapy, adjuvant therapy and recruitment time, patients with BMI < 18.5 kg/m^2^ had an increased risk of death (*HR* = 1.585; 95% *CI:* 1.207–2.082). In addition, patients with higher ALB or PNI or NRI were prone to have lower all-cause mortality (*HR =* 0.716; 95% *CI*: 0.575–0.891; *HR* = 0.793; 95% *CI*: 0.633–0.992; *HR* = 0.588; 95% *CI*: 0.469–0.738, respectively; Table 3). Most importantly, concordances were observed in terms of the discrimination power between PNI (AIC = 4351.984), NRI (AIC = 4335.862) and objective nutritional status indicators ALB (AIC = 4347.161), BMI (AIC = 4346.569), among which NRI had a mildly improved AIC than other three indicators. The predictive performance of model of NRI was consistent with mode of BMI or ALB, or model with both BMI and ALB (AIC = 4341.773). Combining BMI with PNI (AIC = 4345.895) did not improve the discrimination power compared with BMI and PNI alone, or with NRI as results shown in Table 3.

**Table 3:** Multivariate Cox analysis for overall survival of oral cancer patients

Variable	Model 1		Model 2		Model 3		Model 4		Model 5		Model 6	
	*HR* (95%*CI*)	*P*	*HR* (95%*CI*)	*P*	*HR* (95%*CI*)	*P*	*HR* (95%*CI*)	*P*	*HR* (95%*CI*)		*HR* (95%*CI*)	
BMI (kg/m^2^)												
18.5–23.9	1.000								1.000		1.000	
< 18.5	1.585 (1.207,2.082)	0.001							1.530 (1.163,2.013)	0.002	1.546 (1.174,2.034)	0.002
≥ 24.0	0.942 (0.711,1.247)	0.675							0.972 (0.733,1.289)	0.844	0.953 (0.720,1.262)	0.737
ALB (g/l)												
< 40.0			1.000						1.000			
≥ 40.0			0.716 (0.575,0.891)	0.003					0.745 (0.597,0.930)	0.009		
PNI												
< 49.3					1.000						1.000	
≥ 49.3					0.793 (0.633,0.992)	0.043					0.828 (0.659,1.039)	0.104
NRI												
< 97.5							1.000					
≥ 95.5							0.588 (0.469,0.738)	< 0.001				
AIC	4346.569		4347.161		4351.984		4335.862		4341.773		4345.895	
C-index	0.725		0.727		0.721		0.733		0.732		0.727	
Somers’ D	0.450		0.454		0.441		0.465		0.464		0.453	
LR χ^2^	248.820		246.230		241.410		257.530		255.620		251.490	

Note: all adjustment for age, gender, occupation, education level, residence, clinical classification, pathological grading, surgery therapy, adjuvant therapy and recruitment time; LR χ^2^: Likelihood Ratio χ^2^

Additionally, we re-evaluated the association by using disease specific survival as the outcome. After excluding 24 patients who died from other causes instead of oral cancer, and we found the results were consistent with that of overall survival. (Additional file 1: Table S3).

At last, we investigated the association between chemotherapy and prognosis of oral cancer patients with different nutritional status. Adverse association between chemotherapy and survival was observed in patients with a malnutrition status (BMI < 18.5 kg/m^2^, ALB< 40.7 g/l, PNI < 49.3 or NRI < 97.5) after adjusting important clinical features, such as gender, age, clinical classification and surgery therapy when we compare the nutritional indexes’ predictive power between chemotherapy and no chemotherapy patients. However, the adverse association was absent in patients with normal nutritional status, as results shown in Table 4. Since the distribution of variables associated with the disease and patient’s health status is different between patients group with chemotherapy or without chemotherapy, we furtherly measured the predictive power of each of these nutritional variables within each treatment group, separately. Association between nutritional status and prognosis was only observed in oral cancer patients with chemotherapy (Additional file 1: Table S4). These results suggest that nutritional status should be paid more attention in patients with chemotherapy therapy.

**Table 4:** Association between chemotherapy and the prognosis of oral cancer stratified by four nutritional indexes

variables	Chemotherapy ^a^		Chemotherapy ^b^	
	No	Yes	No	Yes
BMI (kg/m^2^)				
18.5–23.9	1.000	0.977 (0.724,1.319)	1.000	0.877 (0.647,1.191)
< 18.5	1.000	1.890 (1.122,3.184)	1.000	2.265 (1.320,3.890)
≥ 24	1.000	1.541 (0.902,2.633)	1.000	1.521 (0.848,2.729)
ALB (g/l)				
< 40.0	1.000	1.585 (1.156,2.173)	1.000	1.424 (1.032,1.965)
≥ 40.0	1.000	0.893 (0.631,1.265)	1.000	0.872 (0.616,1.235)
PNI				
< 49.3	1.000	1.501 (1.116,2.019)	1.000	1.418 (1.051,1.913)
≥ 49.3	1.000	0.893 (0.611,1.307)	1.000	0.821 (0.562,1.199)
NRI				
< 97.5	1.000	1.580 (1.093,2.285)	1.000	1.514 (1.039,2.205)
≥ 95.5	1.000	1.008 (0.745,1.363)	1.000	0.933 (0.688,1.267)

Note: ^a^ adjustment for age, gender, occupation, education level, residence, pathological grading and clinical stage; ^b^ adjustment for age, gender, occupation, education level, residence, pathological grading, clinical stage, surgery therapy and radiotherapy

Since the arsenal of chemotherapy drugs used for head and neck cancer treatment is quite large. So we tried to verify the predictive power of nutritional indexes according to the drug combination used. The most commonly used chemotherapy regimen includes oxaliplatin plus 5-fluorouracil, methotrexate and oxaliplatin plus paclitaxel. And we added a detailed analysis about the prognostic effects of BMI, ALB, PNI and NRI according to different chemotherapy regimen (Additional file 1: Table S5). Only BMI was observed be of statistically significance in patients group with oxaliplatin plus 5-fluorouracil and methotrexate treatment, which may be due to the small sample size for each treatment group.

## Discussion

Factors such as genetic background and molecular biomarkers have been wildly explored in the prognosis of patients with oral cancer [23]. In addition to molecular biomarkers, malnutrition also is a very common characteristic of oral cancer patients and seriously affects their quality of life [24]. Several nutritional markers, including ALB, BMI, PNI and NRI have been developed and used to assess the nutritional status of the cancer patients. In this study we assessed the predictive value of these four most commonly used nutritional markers in a large prospective study. All four markers were significantly associated with OS in oral cancer. Interestingly, malnourished oral cancer patients could have a worse OS from chemotherapy.

BMI is the main tool for nutritional status assessment in clinical. Multiple studies have demonstrated that a low BMI was an independent predictor of a poor prognosis in oral cancer [9, 12]. Our study also confirmed that patients with BMI < 18.5 kg/m^2^ had significantly worse prognosis. In addition to BMI, several epidemiological surveys pointed out that lower preoperative serum albumin was related with an increased rate of postoperative complications (such as wound infection) [5, 6]. Both a decrease of protein intake or consuming nature of the cancer could lead to a decrease in albumin levels [25, 26]. The serum albumin, has been found to be associated with poor survival outcomes in various cancers [10, 13, 27]. Additionally, lymphocyte, as important immune cells, plays a key role in the immune monitoring of tumor cell proliferation, invasion and migration [28, 29]. PNI, which combines albumin and lymphocyte, also has been proved to be significantly related to the prognosis of various tumors including liver cancer, lung cancer and breast cancer et al. [15, 16, 30]. The results of this study also found that ALB and PNI may be useful tools to define the risk of death in oral cancer patients. NRI is another commonly used nutritional indicators which integrated with objective measurements of nutritional status including albumin, height and weights. The study also found that lower levels of NRI was associated with poor survival in oral cancer patients, which is consistent with the previous findings in gastric cancer [17] esophageal squamous cell carcinoma [14] and liver cancer [18].

Currently, few studies made comprehensive evaluation of the prediction performance of BMI, ALB, PNI, and NRI in cancer patients, much less in oral cancer patients. All four markers were significantly associated with OS and showed significant discrimination power in oral cancer. NRI is an indicator that combines weight, height and laboratory data ALB and had mildly better predictive power compared with BMI or ALB alone. In fact, the calculation of NRI and its comparison with the BMI showed that NRI is more sensitive to evaluate risk of malnutrition in oral cancer patients. In our study population, 27.81% of patients (*n* = 388) were malnourished (NRI < 97.5), and 72.19% of the patients (*n* = 1007) had a normal value of NRI. While only 15.34% of patients (*n* = 214) were with BMI < 18.5 kg/m^2^. The results suggested assessment of the nutritional status of oral cancer should not be limited to the collection of weight and height but should also include laboratory data such as ALB. Additionally, consistent results were observed when we compare the predictive performance of NRI with the model including both BMI and ALB, which also suggested that NRI is an appropriate indicator expressing both BMI and ALB. PNI is an indicator which integrates with ALB and lymphocyte. We observed that combination of BMI and PNI did not improve the discrimination power when compared with NRI. This result suggested the limited value of lymphocyte in terms of nutritional status evaluation. Besides, predictive power of BMI and PNI was no better than the performance of BMI or PNI. In fact, when we included both BMI and PNI in the regression model, the contribution of PNI became statistically insignificant, which may because that the contribution of PNI was covered by BMI, or there were underlying correlations between them.

Chemotherapy is an important treatment for oral cancer patients. However, several previous studies found that chemotherapy was not associated with improved prognosis of oral cancer [31]. Hence, whether all patients should be recommended for chemotherapy is still controversial. We observed that chemotherapy was inversely correlated with OS in oral cancer patients with a malnutrition status, and not for those well nourished patients. Actually, up to 86% of cancer patients received chemotherapy suffered from taste impairment or nausea and vomiting [32], which further exacerbates their malnutrition. Therefore, patients with poor nutritional status may be more intolerant of the side effects of chemotherapy, and expect worse prognosis. The results of this study suggest that comprehensive nutritional status assessments may be essential for the design of individualized clinical treatment in oral cancer patients. In our study, a small number of patients (about 69 patients) were submitted to palliative cares which mainly includes nutritional support and pain relief. In addition to palliative care, oral cancer could severely damage the quality of life, especially for advanced patients, and many patients went through emotional disorders such as depression and desperation even committed suicide. Unfortunately, currently there is no consulting service for end of life care in the hospital system, and family and social support have been largely neglected.

## Conclusions

BMI, ALB, PNI and NRI are of prognostic value in oral cancer patients and NRI had the best predictive performance compared with other combinations. Additionally, chemotherapy was inversely related to the prognosis of malnourished patients, indicating that assessment of nutritional status and aggressive nutritional interventions, especially prior to chemotherapy, is crucial for the management of patients affected by oral cancer. Hence, in addition to conventional treatments such as surgery and chemotherapy, more attention should be paid to nutrition support in future treatments, to improve oral cancer patient survival.

## Supplementary information

**Additional file 1:** **Table S1.** Correlation analysis of four nutritional indicators. **Table S2.** Correlation analysis between nutritional indicators and clinically relevant variables (χ^2^ test *P* value). **Table S3.** Multivariate Cox analysis of nutritional index and prognosis of oral cancer for disease-specific survival (DSS). **Table S4.** Association between nutritional indexes and the prognosis of oral cancer in patients group with or without chemotherapy. **Table S5.** Association between nutritional indexes and the prognosis of oral cancer according to chemotherapy regimens.

## Data Availability

The datasets generated and/or analysed during the current study are available from the corresponding author on reasonable request.

## Abbreviations

ALB: Serum albumin
BMI: Body mass index
NRI: Nutritional risk index
PNI: Serum albumin

## References

[1] Zheng CM, Ge MH, Zhang SS, Tan Z, Wang P, Zheng RS, Chen WQ, Xia QM (2015). Oral cavity cancer incidence and mortality in China, 2010. J Cancer Res Ther.

[2] Bray F, Ferlay J, Soerjomataram I, Siegel RL, Torre LA, Jemal A (2018). Global cancer statistics 2018: GLOBOCAN estimates of incidence and mortality worldwide for 36 cancers in 185 countries. CA Cancer J Clin.

[3] Chen F, Lin L, Yan L, Qiu Y, Cai L, He B (2017). Preoperative neutrophil-to-lymphocyte ratio predicts the prognosis of Oral squamous cell carcinoma: a large-sample prospective study. J Oral Maxillofac Surg.

[4] Schwam ZG, Judson BL (2016). Improved prognosis for patients with oral cavity squamous cell carcinoma: analysis of the National Cancer Database 1998-2006. Oral Oncol.

[5] Leung JS, Seto A, Li GK (2017). Association between preoperative nutritional status and postoperative outcome in head and neck Cancer patients. Nutr Cancer.

[6] Danan D, Shonka DC, Selman Y, Chow Z, Smolkin ME, Jameson MJ (2016). Prognostic value of albumin in patients with head and neck cancer. Laryngoscope.

[7] Righini CA, Timi N, Junet P, Bertolo A, Reyt E, Atallah I (2013). Assessment of nutritional status at the time of diagnosis in patients treated for head and neck cancer. Eur Ann Otorhinolaryngol Head Neck Dis.

[8] Kono T, Sakamoto K, Shinden S, Ogawa K (2017). Pre-therapeutic nutritional assessment for predicting severe adverse events in patients with head and neck cancer treated by radiotherapy. Clin Nutr.

[9] Liu SA, Tsai WC, Wong YK, Lin JC, Poon CK, Chao SY, Hsiao YL, Chan MY, Cheng CS, Wang CC (2006). Nutritional factors and survival of patients with oral cancer. Head Neck.

[10] Chandrasinghe PC, Ediriweera DS, Kumarage SK, Deen KI (2013). Pre-operative hypoalbuminaemia predicts poor overall survival in rectal cancer: a retrospective cohort analysis. BMC Clin Pathol.

[11] Saroul N, Pastourel R, Mulliez A, Farigon N, Dupuch V, Mom T, Boirie Y, Gilain L (2018). Which assessment method of malnutrition in head and neck Cancer?. Otolaryngol Head Neck Surg.

[12] Liu F, Chen F, Huang J, Yan L, Liu F, Wu J, Qiu Y, Zheng X, Zhang R, Lin L (2017). Prospective study on factors affecting the prognosis of oral cancer in a Chinese population. Oncotarget..

[13] Uppal S, Al-Niaimi A, Rice LW, Rose SL, Kushner DM, Spencer RJ, Hartenbach E (2013). Preoperative hypoalbuminemia is an independent predictor of poor perioperative outcomes in women undergoing open surgery for gynecologic malignancies. Gynecol Oncol.

[14] Yamana I, Takeno S, Shimaoka H, Yamashita K, Yamada T, Shiwaku H, Hashimoto T, Yamashita Y, Hasegawa S (2018). Geriatric nutritional risk index as a prognostic factor in patients with esophageal squamous cell carcinoma -retrospective cohort study. Int J Surg.

[15] Chan AW, Chan SL, Wong GL, Wong VW, Chong CC, Lai PB, Chan HL, To KF (2015). Prognostic nutritional index (PNI) predicts tumor recurrence of very early/early stage hepatocellular carcinoma after surgical resection. Ann Surg Oncol.

[16] Jin S, Cao S, Xu S, Wang C, Meng Q, Yu Y (2018). Clinical impact of pretreatment prognostic nutritional index (PNI) in small cell lung cancer patients treated with platinum-based chemotherapy. Clin Respir J.

[17] Fujiya K, Kawamura T, Omae K, Makuuchi R, Irino T, Tokunaga M, Tanizawa Y, Bando E, Terashima M (2018). Impact of malnutrition after Gastrectomy for gastric Cancer on long-term survival. Ann Surg Oncol.

[18] Bo Y, Yao M, Zhang L, Bekalo W, Lu W, Lu Q (2015). Preoperative nutritional risk index to predict postoperative survival time in primary liver cancer patients. Asia Pac J Clin Nutr.

[19] Buzby GP, Williford WO, Peterson OL, Crosby LO, Page CP, Reinhardt GF, Mullen JL (1988). A randomized clinical trial of total parenteral nutrition in malnourished surgical patients: the rationale and impact of previous clinical trials and pilot study on protocol design. Am J Clin Nutr.

[20] Onodera T, Goseki N, Kosaki G (1984). Prognostic nutritional index in gastrointestinal surgery of malnourished cancer patients. Nihon Geka Gakkai zasshi.

[21] Buzby GP, Knox LS, Crosby LO, Eisenberg JM, Haakenson CM, McNeal GE, Page CP, Peterson OL, Reinhardt GF, Williford WO (1988). Study protocol: a randomized clinical trial of total parenteral nutrition in malnourished surgical patients. Am J Clin Nutr.

[22] Oh CA, Kim DH, Oh SJ, Choi MG, Noh JH, Sohn TS, Bae JM, Kim S (2012). Nutritional risk index as a predictor of postoperative wound complications after gastrectomy. World J Gastroenterol.

[23] Cervino G, Fiorillo L, Herford AS, Romeo U, Bianchi A, Crimi S, D'Amico C, De Stefano R, Troiano G, Santoro R (2019). Molecular biomarkers related to Oral carcinoma: clinical trial outcome evaluation in a literature review. Dis Markers.

[24] Gellrich NC, Handschel J, Holtmann H, Kruskemper G (2015). Oral cancer malnutrition impacts weight and quality of life. Nutrients..

[25] Shah MA, Capanu M, Soff G, Asmis T, Kelsen DP (2010). Risk factors for developing a new venous thromboembolism in ambulatory patients with non-hematologic malignancies and impact on survival for gastroesophageal malignancies. J Thromb Haemostasis.

[26] Douglas E, McMillan DC (2014). Towards a simple objective framework for the investigation and treatment of cancer cachexia: the Glasgow prognostic score. Cancer Treat Rev.

[27] Ionescu D, Tibrea C, Puia C (2013). Pre-operative hypoalbuminemia in colorectal cancer patients undergoing elective surgery - a major risk factor for postoperative outcome. Chirurgia..

[28] Dunn GP, Bruce AT, Ikeda H, Old LJ, Schreiber RD (2002). Cancer immunoediting: from immunosurveillance to tumor escape. Nat Immunol.

[29] Shoji F, Morodomi Y, Akamine T, Takamori S, Katsura M, Takada K, Suzuki Y, Fujishita T, Okamoto T, Maehara Y (2016). Predictive impact for postoperative recurrence using the preoperative prognostic nutritional index in pathological stage I non-small cell lung cancer. Lung Cancer.

[30] Mohri T, Mohri Y, Shigemori T, Takeuchi K, Itoh Y, Kato T (2016). Impact of prognostic nutritional index on long-term outcomes in patients with breast cancer. World J Surg Oncol.

[31] Chen F, Lin L, Liu F, Yan L, Qiu Y, Wang J, Hu Z, Wu J, Bao X, Lin L (2019). Three prognostic indexes as predictors of response to adjuvant chemoradiotherapy in patients with oral squamous cell carcinoma after radical surgery: a large-scale prospective study. Head Neck..

[32] Silander E, Nyman J, Hammerlid E (2013). An exploration of factors predicting malnutrition in patients with advanced head and neck cancer. Laryngoscope.

