# Supplementary material for: Nutritional assessment and prognosis of oral cancer patients: a large-scale prospective study
Source: BMC Cancer. 2020 Feb 22;20:146. doi: 10.1186/s12885-020-6604-2 (PMC7036168; doi:10.1186/s12885-020-6604-2)
Supplement: Supplementary file 1 — Additional file 1: Table S1. Correlation analysis of four nutritional indicators. Table S2. Correlation analysis between nutritional indicators and clinically relevant variables (χ2 test P value). Table S3. Multivariate Cox analysis of nutritional index and prognosis of oral cancer for disease-specific survival (DSS). Table S4. Association between nutritional indexes and the prognosis of oral cancer in patients group with or without chemotherapy. Table S5. Association between nutritional indexes and the prognosis of oral cancer according to chemotherapy regimens. [file 12885_2020_6604_MOESM1_ESM.docx]

Table S1 Correlation analysis of four nutritional indicators

|  | BMI | ALB | PNI | NRI |
| --- | --- | --- | --- | --- |
| BMI | 1.000 |  |  |  |
| ALB | 0.1719 (<0.001) | 1.000 |  |  |
| PNI | 0.2263 (<0.001) | 0.7988 (<0.001) | 1.000 |  |
| NRI | 0.7638 (<0.001) | 0.7246 (<0.001) | 0.649 (<0.001) | 1.000 |

Table S2 Correlation analysis between nutritional indicators and clinically relevant variables (χ^2^ test *P* value)

|  | BMI (kg/m^2^) | | | |  | ALB (g/l) | | |  | PNI | | |  | NRI | | |
| --- | --- | --- | --- | --- | --- | --- | --- | --- | --- | --- | --- | --- | --- | --- | --- | --- |
|  | 18.5-23.9 | <18.5 | ≥24 | *P* |  | <40.0 | ≥40.0 | *P* |  | <49.3 | ≥49.3 | *P* |  | <97.5 | ≥95.5 | *P* |
| Clinical stage | |  |  | 0.156 |  |  |  | 0.008 |  |  |  | <0.001 |  |  |  | 0.034 |
| Ⅰ | 118(16.30) | 25(14.04) | 35(11.95) |  |  | 63(11.73) | 115(17.48) |  |  | 74(12.25) | 104(17.60) |  |  | 43(12.68) | 135(15.77) |  |
| Ⅱ | 173(23.90) | 45(25.28) | 90(30.72) |  |  | 132(24.58) | 176(26.75) |  |  | 140(23.18) | 168(28.43) |  |  | 76(22.42) | 232(27.10) |  |
| Ⅲ | 135(18.65) | 27(15.17) | 57(19.45) |  |  | 98(18.25) | 121(18.39) |  |  | 107(17.72) | 112(18.95) |  |  | 59(17.40) | 160(18.69) |  |
| Ⅳ | 298(41.16) | 81(45.51) | 111(37.88) |  |  | 244(45.44) | 246(37.39) |  |  | 283(46.85) | 207(35.03) |  |  | 161(47.49) | 329(38.43) |  |
| Pathological grading | |  |  | 0.506 |  |  |  | 0.506 |  |  |  | 0.418 |  |  |  | 0.432 |
| Well | 351(53.10) | 87(53.37) | 120(46.88) |  |  | 248(51.77) | 310(51.58) |  |  | 292(53.28) | 266(50.00) |  |  | 160(53.51) | 398(50.96) |  |
| Moderate | 223(33.74) | 53(32.52) | 99(38.67) |  |  | 160(33.40) | 215(35.77) |  |  | 180(32.85) | 195(36.65) |  |  | 95(31.77) | 280(35.85) |  |
| Poor | 87(13.16) | 23(14.11) | 37(14.45) |  |  | 71(14.83) | 76(12.65) |  |  | 76(13.87) | 71(13.35) |  |  | 44(14.72) | 103(13.19) |  |
| Surgery therapy | |  |  | 0.090 |  |  |  | <0.001 |  |  |  | <0.001 |  |  |  | <0.001 |
| No | 99(12.09) | 29(13.94) | 28(8.33) |  |  | 96(16.22) | 60(7.78) |  |  | 100(14.64) | 56(8.24) |  |  | 62(16.62) | 94(9.49) |  |
| Yes | 720(87.91) | 179(86.06) | 308(91.67) |  |  | 496(83.78) | 711(92.22) |  |  | 583(85.36) | 624(91.76) |  |  | 311(83.38) | 896(90.51) |  |
| Chemotherapy | |  |  | 0.012 |  |  |  | 0.019 |  |  |  | 0.003 |  |  |  | 0.011 |
| No | 499(60.41) | 118(55.66) | 226(67.46) |  |  | 354(59.00) | 489(63.26) |  |  | 400(57.89) | 443(64.96) |  |  | 215(56.58) | 628(63.24) |  |
| Yes | 307(37.17) | 86(40.57) | 107(31.94) |  |  | 226(37.67) | 274(35.45) |  |  | 269(38.93) | 231(33.87) |  |  | 151(39.74) | 349(35.15) |  |
| Radiotherapy |  |  |  | 0.736 |  |  |  | 0.146 |  |  |  | 0.003 |  |  |  | 0.288 |
| No | 498(60.44) | 125(59.81) | 212(63.28) |  |  | 352(58.96) | 483(62.65) |  |  | 391(56.67) | 444(65.49) |  |  | 225(59.68) | 610(61.55) |  |
| Yes | 306(37.14) | 77(36.84) | 117(34.93) |  |  | 226(37.86) | 274(35.54) |  |  | 279(40.43) | 221(32.60) |  |  | 139(36.87) | 361(36.43) |  |

Table S3 Multivariate Cox analysis of nutritional index and prognosis of oral cancer for disease-specific survival (DSS)

| Variable | Model 1 | |  | Model 2 | |  | Model 3 | |  | Model 4 | |  | Model 5 | |  | Model 6 | |
| --- | --- | --- | --- | --- | --- | --- | --- | --- | --- | --- | --- | --- | --- | --- | --- | --- | --- |
|  | *HR* (95%*CI*) | *P* |  | *HR* (95%*CI*) | *P* |  | *HR* (95%*CI*) | *P* |  | *HR* (95%*CI*) | *P* |  | *HR* (95%*CI*) |  |  | *HR* (95%*CI*) |  |
| BMI (kg/m^2^) | |  |  |  |  |  |  |  |  |  |  |  |  |  |  |  |  |
| 18.5-23.9 | 1.000 |  |  |  |  |  |  |  |  |  |  |  | 1.000 |  |  | 1.000 |  |
| <18.5 | 1.549(1.168,2.054) | 0.002 |  |  |  |  |  |  |  |  |  |  | 1.482(1.116,1.968) | 0.007 |  | 1.501(1.130,1.994) | 0.005 |
| ≥24 | 0.998(0.749,1.330) | 0.990 |  |  |  |  |  |  |  |  |  |  | 1.034(0.775,1.379) | 0.821 |  | 1.012(0.759,1.348) | 0.936 |
| ALB (g/l) | |  |  |  |  |  |  |  |  |  |  |  |  |  |  |  |  |
| <40.0 |  |  |  | 1.000 |  |  |  |  |  |  |  |  | 1.000 |  |  |  |  |
| ≥40.0 |  |  |  | 0.699(0.557,0.877) | 0.002 |  |  |  |  |  |  |  | 0.724(0.576,0.911) | 0.006 |  |  |  |
| PNI |  |  |  |  |  |  |  |  |  |  |  |  |  |  |  |  |  |
| <49.3 |  |  |  |  |  |  | 1.000 |  |  |  |  |  |  |  |  | 1.000 |  |
| ≥49.3 |  |  |  |  |  |  | 0.775(0.614,0.978) | 0.032 |  |  |  |  |  |  |  | 0.807(0.638,1.022) | 0.075 |
| NRI |  |  |  |  |  |  |  |  |  |  |  |  |  |  |  |  |  |
| <97.5 |  |  |  |  |  |  |  |  |  | 1.000 |  |  |  |  |  |  |  |
| ≥95.5 |  |  |  |  |  |  |  |  |  | 0.604(0.478,0.763) | <0.001 |  |  |  |  |  |  |

Note: all adjustment for age, gender, occupation, education level, residence, clinical classification, pathological grading, surgery therapy and adjuvant therapy

Table S4 Association between nutritional indexes and the prognosis of oral cancer in patients group with or without chemotherapy

| variable | Chemotherapy therapy | |
| --- | --- | --- |
|  | No | Yes |
| BMI (kg/m^2^) |  |  |
| 18.5-23.9 | 1.000 | 1.000 |
| <18.5 | 0.980(0.634,1.515) | 2.078(1.423,3.035) |
| ≥24 | 0.747(0.503,1.111) | 1.292(0.827,2.017) |
| ALB (g/l) |  |  |
| <40.0 | 1.000 | 1.000 |
| ≥40.0 | 0.764(0.556,1.049) | 0.581(0.417,0.811) |
| PNI |  |  |
| <49.3 | 1.000 | 1.000 |
| ≥49.3 | 0.890(0.647,1.225) | 0.600(0.424,0.848) |
| NRI |  |  |
| <97.5 | 1.000 | 1.000 |
| ≥97.5 | 0.735(0.526,1.026) | 0.510(0.366,0.710) |

Note: all adjustment for age, gender, occupation, education level, residence, pathological grading, clinical stage, surgery therapy and radiotherapy

Table S5 Association between nutritional indexes and the prognosis of oral cancer according to chemotherapy regimens

| variables | Oxaliplatin+5-fluorouracil (n=70) | |  | Methotrexate (n=47) | |  | Oxaliplatin+Paclitaxel (n=24) | |  | Others (n=75) | |
| --- | --- | --- | --- | --- | --- | --- | --- | --- | --- | --- | --- |
|  | *HR* (95%*CI*) | *HR* (95%*CI*) ^a^ |  | *HR* (95%*CI*) | *HR* (95%*CI*) ^a^ |  | *HR* (95%*CI*) | *HR* (95%*CI*) ^a^ |  | *HR* (95%*CI*) | *HR* (95%*CI*) ^a^ |
| BMI (kg/m^2^) | |  |  |  |  |  |  |  |  |  |  |
| 18.5-23.9 | 1.000 | 1.000 |  | 1.000 | 1.000 |  | 1.000 | 1.000 |  | 1.000 | 1.000 |
| <18.5 | 2.943(0.958,9.042) | 5.780(1.193,28.001) |  | 2.961(1.109,7.908) | 3.653(1.103,12.097) |  | 3.858(0.694,21.455) | 3.044(0.046,203.478) |  | 1.331(0.482,3.676) | 1.964(0.618,6.242) |
| ≥24 | 1.971(0.593,6.552) | 2.639(0.633,10.993) |  | 1.918(0.399,9.214) | 18.176(1.337,247.132) |  | 0.483(0.054,4.332) | 0.483(0.023,10.174) |  | 0.643(0.233,1.774) | 1.066(0.323,3.516) |
| ALB (g/L) |  |  |  |  |  |  |  |  |  |  |  |
| <40.0 | 1.000 | 1.000 |  | 1.000 | 1.000 |  | 1.000 | 1.000 |  | 1.000 | 1.000 |
| ≥40.0 | 0.659(0.254,1.712) | 0.778(0.238,2.542) |  | 0.976(0.383,2.490) | 1.109(0.306,4.019) |  | 0.591(0.132,2.641) | 0.343(0.012,10.173) |  | 0.378(0.166,0.861) | 0.298(0.107,0.832) |
| PNI |  |  |  |  |  |  |  |  |  |  |  |
| <49.3 | 1.000 | 1.000 |  | 1.000 | 1.000 |  | 1.000 | 1.000 |  | 1.000 | 1.000 |
| ≥49.3 | 0.607(0.229,1.607) | 0.714(0.247,2.063) |  | 0.664(0.257,1.716) | 0.629(0.174,2.269) |  | 1.573(0.352,7.033) | 2.078(0.078,55.549) |  | 0.668(0.288,1.548) | 0.652(0.250,1.701) |
| NRI |  |  |  |  |  |  |  |  |  |  |  |
| <97.5 | 1.000 | 1.000 |  | 1.000 | 1.000 |  | 1.000 |  |  | 1.000 | 1.000 |
| ≥97.5 | 0.365(0.141,0.948) | 0.457(0.138,1.514) |  | 0.453(0.170,1.212) | 0.366(0.070,1.906) |  | 0.303(0.068,1.358) | - |  | 0.648(0.279,1.502) | 0.514(0.181,1.454) |

Note: ^a^ Adjusted for age, gender, occupation, education level, residence, pathological grading, clinical stage, surgery therapy and radiotherapy
